# Editorial: Bariatric surgery—its influence on the development, diagnosis, and treatment of tumors

**DOI:** 10.3389/fsurg.2022.1110401

**Published:** 2022-12-23

**Authors:** Marcel André Schneider, Sivamainthan Vithiananthan, Daniel Gero

**Affiliations:** ^1^ Department of Surgery and Transplantation, University Hospital Zurich, University of Zurich, Zurich, Switzerland; ^2^ Department of Surgery, Cambridge Health Alliance & Harvard, T.H. Chan School of Public Health Cambridge, Cambridge, MA, United States

**Keywords:** bariatric (weight loss) surgery, metabolic surgery, visceral surgery, obesity, cancer

**Editorial on the Research Topic** Bariatric surgery—its influence on the development, diagnosis, and treatment of tumors

Metabolic bariatric surgery (MBS) has been shown to be safe (1, 2), effective, and durable in the treatment of clinically severe obesity and its co-morbidities (3). Laparoscopic gastric bypass and laparoscopic sleeve gastrectomy are currently the most frequently performed and investigated bariatric procedures that lead to comparable long-term weight loss (4, 5). With the ongoing rise in the prevalence of obesity, the number of bariatric procedures is expected to increase steadily over the next decades (6).

The influence of obesity on the development of many tumor types (e.g., esophagogastric-junction cancer (7), metabolic-associated fatty liver disease (MAFLD)-related hepatocellular carcinoma, and endometrial cancer) is well documented (8). While MBS is a highly effective method to reduce the disease burden of obesity, there is a lack of investigation into how postbariatric physiology and weight loss influence tumor development, diagnosis, and treatment outcomes (9).

Evidence stemming from large-scale observational studies indicated reduced overall cancer incidence in patients undergoing bariatric surgery (10), and an array of recent studies confirmed reduced incidence of obesity related cancers after MBS (11, 12), even for cancer types not associated with obesity, such as lung cancer (13, 14). In contrast, certain bariatric techniques might increase the risk of acid- or biliary reflux disease, potentially leading to dysplastic changes of the distal esophageal mucosa (15). Bariatric anatomy may even hinder the conventional diagnosis of malignancies of the foregut by jeopardizing endoscopic access to the duodenum, the biliary system, and to the main pancreatic duct (16).

The aim of the current Research Topic was therefore to cover promising, recent, and novel research in the overlapping clinical field of oncology and MBS as well as endocrinology, gastroenterology, metabolism, and nutrition. Our goal was to gather a collection of original or review articles that address current and future challenges that arise in the setting of tumor development, diagnosis and treatment after MBS, and enable increased insight into the interdisciplinary approach to oncology in patients with obesity. Overall, our Research Topic has sparked light interest; we have assembled four manuscripts, three original research articles and one narrative review.

The assessment of systemic inflammation, especially the changes of the neutrophil-to-lymphocyte ratio (NLR) following laparoscopic sleeve gastrectomy, was the focus of two original research articles. The NLR in the peripheral blood is a well-known indicator of subclinical inflammation and has been associated with poorer prognoses in ischemic coronary disease, heart failure, peripheral vascular disease, and patients with obesity (17). Zhou et al. retrospectively analyzed the laboratory values of 72 patients and confirmed that at 7 months post operation, MBS leads to a significant decrease in inflammatory markers from the preoperative baseline (NLR: 2.4 ± 1.59 vs. 1.7 ± 0.86; C-reactive protein: 5.6 ± 3.17 vs. 2.1 ± 2.35 mg/L, both *p* < 0.001). A subgroup analysis showed that females showed a higher decrease in NLR than male patients [OR = 3.14 (95%CI: 1.112–8.870); *p* = 0.031]. Chi et al. presented changes in the NLR in 100 patients and reported a significant decrease already at 3 months post operation (2.21 vs. 1.78, *p* = 0.005). While these data confidently report optimization of the systemic inflammatory state after MBS, the underlying physiologic mechanism and its role on oncogenesis is yet to be investigated.

Ross et al. published a state-of-the art review focusing on the relationship between MBS and endometrial cancer. They reported that among different types of cancers, endometrial cancer had the strongest association with obesity, and weight loss may lead to a regression of premalignant pathology. The authors gathered evidence supporting that a decreased incidence of hormonally responsive cancers (endometrial, breast, and prostate cancer) was associated with MBS. Therefore, they pledged for well-designed, prospective, and mechanistic studies to clarify the most appropriate candidates and time for MBS in patients with obesity and early-stage endometrial cancer.

Mehdorn et al. performed a retrospective double center study in patients with obesity and MAFLD-related Child A liver cirrhosis who underwent MBS. They reported ambiguous outcomes, with some patients presenting a postbariatric aggravation of their liver function and others showing improvement that resulted in un-listing from the liver transplantation waiting list. Nevertheless, according to the 2022 IFSO/ASMBS guidelines, with parsimonious patient selection and a multidisciplinary surgical pre-habilitation pathway, MBS has the potential to improve the candidacy for transplantation in patients with an end-stage organ disease (3).

Taken together, these findings highlight the role of MBS in the reduction of systemic inflammation and, consequently, the risk of cancer, but they also showcase the need for more extensive basic and clinical research in the field. The main limitation of our Research Topic is the lack of original research publication in the field of bariatric oncology, leaving the primary aims of the Research Topic unmet. However, we are confident that all the selected studies in our Research Topic pave the way for further scientific interest aiming to better diagnose, prevent, and treat cancer in patients with obesity and/or history of MBS.
